# Climate projections for glacier change modelling over the Himalayas

**DOI:** 10.1002/joc.6298

**Published:** 2019-12-25

**Authors:** Martin W. Jury, Thomas Mendlik, Satyanarayana Tani, Heimo Truhetz, Douglas Maraun, Walter W. Immerzeel, Arthur F. Lutz

**Affiliations:** ^1^ Wegener Center for Climate and Global Change (WEGC) University of Graz Graz Austria; ^2^ Institute of Microwave and Photonic Engineering Graz University of Technology Graz Austria; ^3^ Department of Physical Geography Utrecht University Utrecht The Netherlands; ^4^ Future Water Wageningen The Netherlands

## Abstract

Glaciers are of key importance to freshwater supplies in the Himalayan region. Their growth or decline is among other factors determined by an interaction of 2‐m air temperature (TAS) and precipitation rate (PR) and thereof derived positive degree days (PDD) and snow and ice accumulation (SAC). To investigate determining factors in climate projections, we use a model ensemble consisting of 36 CMIP5 general circulation models (GCMs) and 13 regional climate models (RCMs) of two Asian CORDEX domains for two different representative concentration pathways (RCP4.5 and RCP8.5). First, we downsize the ensemble in respect to the models' ability to correctly reproduce dominant circulation patterns (i.e., the Indian summer monsoon [ISM] and western disturbances [WDs]) as well as elevation‐dependent trend signals in winter. Within this evaluation, a newly produced data set for the Indus, Ganges and Brahmaputra catchments is used as observational data. The reanalyses WFDEI, ERA‐Interim, NCEP/NCAR and JRA‐55 are used to further account for observational uncertainty. In a next step, remaining TAS and PR data are bias corrected applying a new bias adjustment method, scale distribution mapping, and subsequently PDD and SAC computed. Finally, we identify and quantify projected climate change effects. Until the end of the century, the ensemble indicates a rise of PDD, especially during summer and for lower altitudes. Also TAS is rising, though the highest increases are shown for higher altitudes and between December and April (DJFMA). PRs connected to the ISM are projected to robustly increase, while signals for PR changes during DJFMA show a higher level of uncertainty and spatial heterogeneity. However, a robust decline in solid precipitation is projected over our research domain, with the exception of a small area in the high mountain Indus catchment where no clear signal emerges.

## Graphical abstract

Until the end of the century, the ensemble indicates a rise of positive degree days, especially during summer and for lower altitudes. Precipitation (PR) connected to the Indian summer monsoon are projected to robustly increase, while signals for PR changes during DJFMA show a higher level of uncertainty and spatial heterogeneity. Solid precipitation is robustly projected to decline except for some areas in the high mountain Indus catchment. Changes in the annual cycle of positive degree days (PDD, top row) and snow and ice accumulation (SAC, bottom row) for RCP4.5 (first and third column) and RCP8.5 (second and fourth column) until the end of the century (1981–2010 to 2071–2100). The black line indicates the ensemble mean. Dashed lines show the 25th and 75th percentile (grey shaded area), dotted lines show the 5th and 95th percentile (light‐grey shaded area). The number in the top right corner of each plot indicates the number of included climate models.

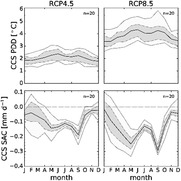

## 1 INTRODUCTION

The Himalayan region, sometimes referred to as the water tower of Asia or High Mountain Asia (HMA), is home to the highest mountains on our planet. Located between 28° and 38°N latitude runoff from the Hindu Kush, Karakoram and southern Himalaya (HKKH) feed three major rivers, the Indus, Ganga and Brahmaputra, whose catchments are in the focus of this study. The water generated in the HKKH has varying importance for the downstream areas. In comparison to the Ganga and the Brahmaputra, the Indus basin is more dependent on upstream water resources, because of its very arid downstream climate, westerly influenced precipitation regimes and large glacier systems (Immerzeel and Bierkens, 2012; Schaner *et al*., 2012; Lutz *et al*., 2014). Furthermore, the Indus basin has the world's largest irrigation scheme (Jain *et al*., 2007), but also the Ganga and Brahmaputra river basins have extensive areas of irrigated agriculture. All three river basins have large populations, which are growing at high rates and will demand more water and energy in the future (Wijngaard *et al*., 2018).

With a warming climate hydrological systems in high elevations are expected to experience massive changes (e.g., Beniston, 2003). In addition, there are a number of indications that air masses at higher altitudes warm faster compared to lower altitudes, a process termed elevation‐dependent warming (EDW, Pepin *et al*., 2015). Because glaciers serve as water reserves for lower regions they are of key importance for the water balance of a region. Under a warming climate, rising temperatures trigger and accelerate melt of glaciers while changing precipitation regimes may even increase the mass intake of glaciers if they lead to increases in solid precipitation.

Two major meteorologic phenomena influence the southern Himalayan weather regimes controlling the water balance of the Indus, Ganga and Brahmaputra rivers: the Indian summer monsoon (ISM) and Western Disturbances (WDs). The ISM brings large amounts of precipitation between June and September (JJAS), while WDs introduce cold air and precipitation especially to the Hindu Kush and Karakoram mainly during winter and early spring often in the form of snow (between December and April [DJFMA]), thereby providing an important source of mass intake for glaciers.

The ISM is formed by winds transporting moist air from the Indian Ocean over the Indian subcontinent. During the monsoon season (JJAS) the moist air propagates northward and is deflected to the West by the Himalayas. The strongest precipitation occurs at the south‐eastern flanks of the Himalayas while the Hindu Kush and Karakoram are experiencing less precipitation intake (see also Figure 3 in Subsection 3.1). Under the influence of increasing greenhouse gases, general circulation models (GCMs) project an increase of the monsoon precipitation with a simultaneous weakening of the monsoon circulation possibly due to a weakening of land–sea temperature differences (e.g., May, 2011; Chaturvedi *et al*., 2012; Christensen *et al*., 2013). Further, there are indications for a positive trend in extreme precipitation as well as a projected increase in day‐to‐day rainfall variability for the high emission scenario during the monsoon period (Chaturvedi *et al*., 2012; Menon *et al*., 2013).

WDs originate from extratropical storms developing in the Mediterranean which are subsequently transported eastward via the mid‐latitude subtropical jet and interact with the Indian winter monsoon (Dimri *et al*., 2015). While precipitation from the stochastic WDs is weaker than monsoon precipitation, it has a major impact on water supply and agriculture. GCMs from the phase 3 of the Coupled Model Intercomparison Project (CMIP3) indicate an increase in storm activity while the more recent CMIP5 models suggest the opposite together with a poleward shift of the storm track (Chang *et al*., 2012).

State‐of‐the‐art climate models have difficulties simulating regional patterns of monsoon precipitation, while studies on their performance for WDs in the Himalayas are scarce (Turner and Annamalai, 2012; Asharaf and Ahrens, 2015; Sharmila *et al*., 2015). Nevertheless, numerous studies have shown an improvement of regional to local processes in regional climate models (RCMs) often attributed to improved model physics and finer resolution (Prein *et al*., 2015; Torma *et al*., 2015; Giorgi *et al*., 2016). Especially in areas characterized by a complex orography like mountainous regions, RCMs achieve better results due to a higher resolved topography (Jones *et al*., 1995; Di Luca *et al*., 2013; Kotlarski *et al*., 2015). In particular, improvements have been shown for larger‐scale phenomena like WDs and the south Asian monsoon with higher resolution (e.g., Dimri *et al*., 2006, 2013; Karmacharya *et al*., 2016). Krishnan *et al*. (2018) showed that increasing temperatures and an associated strengthening of the east–west temperature gradient across the Tibetan Plateau lead to increases in DJFMA precipitation associated with WD activity using a variable grid atmospheric GCM focusing on the bigger Himalayan region.

One key process in mountainous regions is elevation‐dependent warming (EDW, Yan and Liu, 2014; Pepin *et al*., 2015). The snow and ice albedo feedback is considered to be one major driver of EDW. With a warming atmosphere, the melting of snow and ice in higher elevations decreases the albedo and thereby leads to an added warming compared to lower lying areas. Because of sparse station data at high altitudes, trend studies in mountain regions based on observations are rare, though EDW was shown to be highest in December–February (DJF) over the Tibetan Plateau (Yan and Liu, 2014). While state‐of‐the‐art GCMs are capable of producing EDW signals (Palazzi *et al*., 2017; Rangwala *et al*., 2016), RCMs are generally expected to reproduce EDW better than GCMs due to their better resolved orography and hence better representation of higher elevations (Giorgi *et al*., 1997; Kotlarski *et al*., 2015).

Here, we undertake the effort to provide bias‐corrected climate scenarios over the HKKH with a particular aim to subsequently provide an adequate data set to the hydrologic climate change impact modelling community. To do this, we combine state‐of‐the‐art climate simulations from both, GCMs and RCMs (CMIP5 and Coordinated Regional Climate Downscaling Experiment [CORDEX] models, respectively; Taylor *et al*., 2012; Giorgi *et al*., 2009; Evans, 2011, see Subsection 2.3), with a model evaluation (Section 3), selecting only models which are able to reproduce key weather phenomena and a simplified form of EDW. Further, we correct the remaining models using a novel statistical bias adjustment method (see Subsection 2.4), which preserves the projected climate change signal of the climate models. This is an improvement to other studies applying variance correcting bias adjustment like quantile mapping. Also, we use a new observational data set (see Subsection 2.2), to derive robust detailed climate change projections until the end of the century for the Indus, Ganga and Brahmaputra river basins. Finally, we estimate climate change effects and their uncertainties on regional meteorological drivers that are dominating the behaviour of glaciers in general (Section 4.1).

## 2 DATA AND METHODS

### 2.1 Variables and indices

A glacier's mass balance is the result of the total ablation and the total snow accumulation over the glacier surface. In this paper, we focus on changes in 2‐m air temperature (TAS) and precipitation rate (PR) caused by global climate change until the end of the century, and additionally on indices important for hydrologic glacier modelling, that is, positive degree days (PDD) and snow and ice accumulation (SAC). Many glacier models use air temperature as a proxy for glacier melt in the form of a positive degree day model. In such models, the total melt is the time accumulated number of TAS degrees above zero (PDD) multiplied by a degree day factor. In our study, we use PDD as a proxy for the total melt of a glacier. SAC is the total accumulated amount of snow fall over the glacier area and is an indicator of potential changes on the supply side. Throughout this paper we have derived SAC values from PR values conditioned on TAS below 1.5°C.

### 2.2 Observational data sets

**Figure 1:**
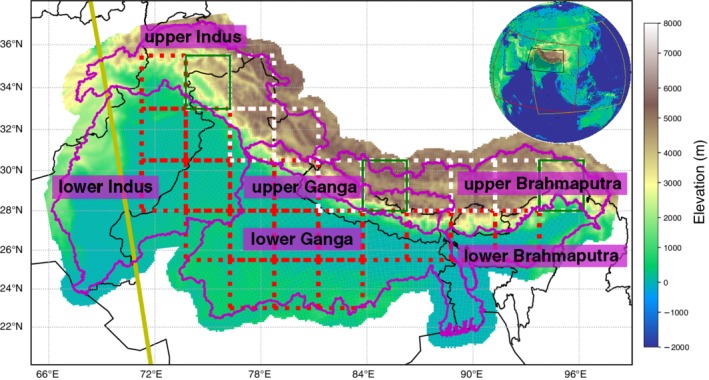
Orography of the observational IGB‐data set with a resolution of 10 km × 10 km. The global plot indicates the domains of EA‐CORDEX (yellow) and SA‐CORDEX (red) as well as the research domain (black square). White and red boxes indicate the evaluation grid boxes. Green boxes indicate the three regions used to present differences in the annual PR patterns (see Figure 3). The purple lines indicate the upstream and downstream river basins of the Indus, Ganga and Brahmaputra rivers

As observational data, we use a novel data set of daily air temperature and precipitation fields at 10 × 10 km resolution covering 1981–2010 at a daily time step, developed for the Indus, Ganga and Brahmaputra river basins (IGB, Lutz *et al*., 2018, see Figure 1). This data set is based on the Watch Forcing ERA‐Interim (WFDEI) data set (Weedon *et al*., 2011, 2014) and was generated in the following way: For the upstream basins, the raw temperature data were spatially interpolated from a resolution of 0.5° × 0.5° to a resolution of 1 × 1 km, and subsequently downscaled using a 1 × 1 km digital elevation model (DEM) derived from the shuttle radar topography mission (SRTM) DEM (Farr *et al*., 2007) and vertical monthly temperature lapse rates. The downscaled temperature data were bias‐corrected to the observations of 40 meteorological stations located in the upstream basins. In addition, a temperature bias‐correction is conducted, by capping the average annual glacier ablation to a maximum plausible value (Immerzeel *et al*., 2012; Ragettli *et al*., 2015), to avoid unrealistic high temperatures at high altitudes. The raw precipitation data were similarly spatially interpolated, and subsequently corrected for the underestimate of high‐altitude precipitation by using geodetic mass balance data (Gardelle *et al*., 2013) as a proxy to reconstruct precipitation amounts, according to the method described by Immerzeel *et al*. (2015). Finally, the corrected 1 × 1 km temperature and precipitation data sets were aggregated to a resolution of 10 × 10 km. For the downstream basin, no bias corrections have been applied to WFDEI data. Also, air temperature data is spatially interpolated from 0.5° × 0.5° resolution to 10 × 10 km and downscaled using a DEM and vertical temperature lapse rates, whereas precipitation data where only spatially interpolated to the 10 × 10 km resolution. The meteorological data required for the bias‐correction of the temperature data sets were obtained from 40 meteorological stations that are acquired through the Nepal Department of Hydrology and Meteorology (DHM), Pakistan Meteorological Department (PMD) and the Pakistan Water and Power Development Authority (WAPDA).

Different observations may lead to different conclusions, especially over areas with sparse direct measurements of considered properties. To account for observational uncertainty during the model evaluation (Collins *et al*., 2013, see Section 3), we further considered several reanalysis datasets: the European Centre for Medium‐Range Weather Forecasts (ECMWF) Interim reanalysis (ERA‐Interim), Japanese 55‐year reanalysis (JRA55) and 40‐year National Centers for Environmental Prediction/National Center for Atmospheric Research reanalyses (NCEP/NCAR) in our analyses. Because IGB is based upon the WFDEI data set using global precipitation climatology centre (GPCC) data to correct precipitation rates, we included also this data set in our evaluations. Asian Precipitation Highly Resolved Observational Data Integration Towards Evaluation (APHRODITE) has been shown to seriously underestimate precipitation over the HKKH (Immerzeel *et al*., 2015, see also Figure 3), making it unsuitable for glacier impact studies. Nevertheless, we included it for the period of 1980–2007 for demonstrative reasons, while we did not use it in the model evaluation (see Supporting Information, Figures S1,3 and 4 in Section 3). An overview of all used observational data sets is given in Table 1.

**Table 1:** Overview of the used observational datasets

Name	Institution	Spatial res.	Reference
IGB	FutureWater, HI‐AWARE consortium	10 km × 10 km	Lutz *et al*. (2018)
WFDEI GPCC	Water and Global Change (WATCH) Project, Europe	0.5° × 0.5°	Weedon *et al*. (2011), Weedon *et al*. (2014), Schneider *et al*. (2013)
ERA‐interim	European Centre for Medium‐Range Weather Forecasts, Europe	0.75° × 0.75°	Dee *et al*. (2011)
JRA55	Japan Meteorological Agency, Japan	1.25° × 1.25°	Kobayashi *et al*. (2015) and Harada *et al*. (2016)
NCEP/NCAR	National Centers for Environmental Prediction/National Center for Atmospheric Research Reanalyses, USA	2.5° × 2.5°	Kalnay *et al*. (1996)
APHRODITE	Japan Meteorological Agency, Japan	0.25° × 0.25°	Yasutomi *et al*. (2011) and Yatagai *et al*. (2012)

### 2.3 Climate models

Our climate model ensemble consists of GCMs from the CMIP5 (Taylor *et al*., 2012) and RCMs from two Coordinated Regional Climate Downscaling Experiment (CORDEX) domains over the HKKH region: South‐Asia CORDEX (SA‐CORDEX) and East‐Asia CORDEX (EA‐CORDEX) (Giorgi *et al*., 2009; Evans, 2011). We use data of two emission scenario experiments, the representative concentration pathways (RCPs) 4.5 and 8.5 until the end of the century (2005–2100). For the observational period (1981–2010) we use data from the historical experiment (until 2005) and fill up the remaining 5 years with data of RCP4.5 for every model (RCP8.5 if no data for RCP4.5 was available). Thirty‐six CMIP5 GCMs have been included in our initial ensemble. Thirty‐four of which provided climate projections under RCP4.5 and 33 under RCP8.5. Additionally 13 RCMs from the two CORDEX regions with a horizontal resolution of 50 km have been included. Eight RCMs^1^ stem from the SA‐CORDEX ensemble (eight under RCP4.5 and six under RCP8.5) and five RCMs from the SA‐CORDEX ensemble (four under RCP4.5 and three under RCP8.5). The included RCMs dynamically downscaled driving data provided by six different CMIP5 GCMs in the case of SA‐CORDEX (three RCM runs downscaled data from MPI‐ESM‐LR and one respective RCM run data from GFDL‐CM3, ACCESS1‐0, CNRM‐CM5, EC‐EARTH and NorESM1‐M), and two CMIP5 GCMs over the EA‐CORDEX domain (four RCM runs downscaled data from HadGEM2‐AO and one data from EC‐EARTH). In total eight different institutes utilized nine different RCMs (MM5, WRF, RegCM4, HIRHAM5, HadGEM3‐RA, REMO2009, RCA4, CCLM and the mentioned CCAM‐1391M) to dynamically downscale climate information over our research domain. In total we included 49 climate model runs (see Table 2 for an overview).

**Table 2:** Summary table of the model evaluation

Model	*r*_TAS_	*r*_PR_	∆TAS	Model	*r*_TAS_	*r*_PR_	∆TAS
IGB	0.79	0.68	0.32	GFDL‐ESM2G r1i1p1	0.73	0.53	−0.01
WFDEI_GPCC	0.72	0.78	0.32	GFDL‐ESM2M r1i1p1	0.74	0.58	−0.09
JRA‐55	0.90	0.86	0.07	GISS‐E2‐H r6i1p3	0.67	0.39	0.26
NCEP/NCAR	0.82	0.79	0.29	GISS‐E2‐R r6i1p1	0.69	0.21	−0.07
ERA‐Int	0.72	0.68	0.18	HadGEM2‐AO r1i1p1	0.86	0.71	−0.12
ACCESS1‐0 r1i1p1	0.86	0.72	0.23	EA NIMR HadGEM3‐RA	0.91	0.79	−0.09
SA CSIRO CCAM‐1391M	0.86	0.51	−0.09	EA SNU MM5	0.86	0.72	0.63
ACCESS1‐3 r1i1p1	0.86	0.61	0.25	EA SNU WRF	0.88	0.73	0.46
bcc‐csm1‐1 r1i1p1	0.64	0.00	−0.12	EA KNU RegCM4	0.86	0.81	0.22
bcc‐csm1‐1‐m r1i1p1	0.85	0.60	0.02	HadGEM2‐CC r1i1p1	0.86	0.69	0.29
BNU‐ESM r1i1p1	0.67	0.13	0.02	HadGEM2‐ES r1i1p1	0.87	0.72	0.34
CanESM2 r1i1p1	0.74	0.56	0.40	inmcm4 r1i1p1	0.78	0.60	−0.3
CCSM4 r1i1p1	0.87	0.71	0.23	IPSL‐CM5A‐LR r1i1p1	0.72	0.03	−0.02
CESM1‐BGC r1i1p1	0.87	0.75	0.14	IPSL‐CM5A‐MR r1i1p1	0.85	0.50	0.14
CESM1‐CAM5 r1i1p1	0.87	0.76	0.22	IPSL‐CM5B‐LR r1i1p1	0.73	−0.38	−0.02
CMCC‐CESM r1i1p1	0.35	0.02	0.07	MIROC‐ESM r1i1p1	0.54	0.19	0.19
CMCC‐CM r1i1p1	0.89	0.78	0.12	MIROC‐ESM‐CHEM r1i1p1	0.52	0.19	0.31
CMCC‐CMS r1i1p1	0.82	0.70	0.19	MIROC5 r1i1p1	0.84	0.77	0.16
CNRM‐CM5 r1i1p1	0.83	0.79	0.18	MPI‐ESM‐LR r1i1p1	0.81	0.67	0.22
SA CSIRO CCAM‐1391M	0.85	0.53	−0.15	SA MPI‐CSC REMO2009	0.90	0.78	0.11
CSIRO‐Mk3‐6‐0 r1i1p1	0.73	0.42	0.45	SA IAU CCLM	0.86	0.83	−0.02
CSIRO‐Mk3L‐1‐2 r1i2p1	0.38	−0.18	0.15	SA CSIRO CCAM‐1391M	0.85	0.49	0.06
EC‐EARTH r12i1p1	0.88	0.81	−0.21	MPI‐ESM‐MR r1i1p1	0.81	0.70	0.07
SA SMHI RCA4	0.91	0.79	−0.05	MRI‐CGCM3 r1i1p1	0.85	0.67	0.23
EA r3i1p1 DMI HIRHAM5	0.90	0.73	0.10	MRI‐ESM1 r1i1p1	0.84	0.66	0.25
GFDL‐CM3 r1i1p1	0.85	0.62	0.19	NorESM1‐M r1i1p1	0.67	0.57	0.09
SA CSIRO CCAM‐1391M	0.85	0.51	−0.43	SA CSIRO CCAM‐1391M	0.85	0.50	0.14

Columns denote the modelname, the lowest Pearson correlation for TAS (*r*_TAS_) and PR (*r*_PR_) as well as trend differences between lower grid points (< 2000 m a.s.l.) and grid points above 3,000 m a.s.l. for DJF in ^°^C (ΔTAS). Criteria that led to the exclusion of one particular model are indicated by grey shadings. RCM model names are indented and located under their respective driving CMIP5 GCM modelname and inform about the underlying CORDEX ensemble (SA‐CORDEX: SA, EA‐CORDEX: EA).

### 2.4 Bias correction

Climate models, both GCMs and RCMs, feature systematic biases, while assessments of climate change impacts require realistic estimates of possible changes to come. Bias correction is one way to bridge the gap between biased model output and data demands of the impact modelling community (e.g., Maraun, 2016). It is important to note that bias correction is uninformed of underlying physical processes and their representation in the climate models. Subsequently process misrepresentations may lead to biases in the corrected data itself (e.g., Addor *et al*., 2016). To control for misrepresentations in the raw model data and to ensure that our ensemble consists only of models which are able to reproduce basic key characteristics of the climate system relevant for climate change impacts on glaciers we conducted a model evaluation (see next section). We used a trend‐preserving bias adjustment method, scale distribution mapping (SDM, Switanek *et al*., 2017), which allows us to preserve beforehand asserted elevation‐dependent trend signals in the models (see Subsection 3.2).

## 3 PROCESS‐BASED MODEL EVALUATION

Differences in regional projections of climate change are to a certain extent attributable to a miss‐positioning or miss‐timing of weather phenomena in climate models or a corruption of important physical processes that may alter climate change in different aspects (e.g., Hall, 2014; Shepherd, 2014). One way to overcome this limitation is to evaluate models in terms of their ability to reproduce relevant weather phenomena and key processes and exclude those who show shortcomings in that respect. We argue that models missing key processes relevant for a region in the observable past are unlikely to provide credible projections of future climate (e.g., Knutti, 2010; McSweeney *et al*., 2012; Hall, 2014). In particular, we evaluate the models' ability to reproduce the mean timing and spatial distribution of the ISM and WDs as well as a simplified form of observable EDW.

### 3.1 Circulation‐based phenomena

Weather patterns are diverse over the HKKH. The quality of representation of important weather systems varies in state‐of‐the‐art climate models. Additionally, the applied bias adjustment does not correct biases in the representation of atmospheric processes like circulation patterns. Both arguments demanded a thorough evaluation of the climate models as precipitation regimes in this region are majorly driven by two distinct processes, the Indian summer monsoon (ISM) and western disturbances (WDs).

Several studies evaluate the representation of monsoon systems in state‐of‐the‐art climate models, while evaluation of WDs is rare. All of them have been focusing on different aspects of one weather phenomena in question, most of the time accounting for a multitude of criteria and focusing on a rather large domain (e.g., Chang *et al*., 2012; Ridley *et al*., 2013; Sperber *et al*., 2013; Lee and Wang, 2014; Ghimire *et al*., 2018; Sharmila *et al*., 2015).

**Figure 2:**
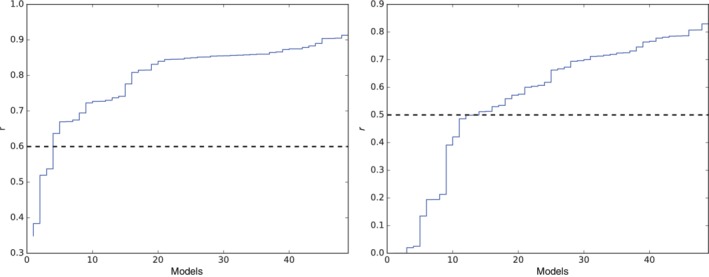
Lowest values of *r* for TAS (left) and PR (right) of all evaluated models. The rejection criterion is marked by the dashed black line

Here, we calculated Pearson correlation coefficients of TAS and PR to our observations (IGB) in temporal, spatial and combined dimensions on the southern slopes of the Himalayas to capture model performance for both phenomena in one step. In doing so, we do not control for the circulation systems directly, but we ensure that only those models that are able to reproduce connected tangible surface impacts remain in our model ensemble. In comparison to spatial and temporal patterns, absolute amplitudes of, for example, PRs are of minor importance during our model evaluation, as they are corrected by the applied bias adjustment. All models of the ensemble have been conservatively regridded to a 2.5° × 2.5° regular grid. For every grid point we derived the 30 year monthly means for our historical period (*∅* January 1, 1981–December 31, 2010, giving us 12 values per grid point), and in order to capture representations of the mentioned weather phenomena in a higher mountain environment, only grid points above 3,000 m above sea level (a.s.l.) are included in the analysis (c.f. white squares in Figure 1). Subsequently, we calculated Pearson correlations coefficients of all models to our observational data sets for each grid point over time (referred to as temporal correlations hereafter), for each month over the evaluation grid points (referred to as spatial correlations hereafter) and for all data points combined (referred to as combined correlations hereafter). The singe correlations coefficients over the temporal and spatial dimensions have been aggregated using Fisher z‐transformations (see Tables S1 and S2). To account for the observational uncertainty (Collins *et al*., 2013), correlations were also calculated with every reanalysis as reference (WFDEI‐GPCC, ERA‐Interim, JRA55 and NCEP/NCAR). For TAS and PR the lowest correlation coefficient *r* of every model to any observational data set was consulted to remove poor performing models from the ensemble. The limit has been set to 0.6 for TAS and to 0.5 for PR, which is about 1.5 times the observational uncertainty (i.e., the lowest correlation among the observational data sets). Below this threshold a strong decline in model performance is observed (see Figure 2).

#### 3.1.1 Selection of climate models based on the representation of large‐scale climate phenomena

Almost all climate models are able to reproduce the annual cycle of temperature on the evaluation grid (see Figure S1). For both, observational and climate model data, spatial correlations show the lowest correlations, while temporal correlations are not lower than 0.97 (c.f. Table S1). Between our observational data sets mostly high correlations exist, with the lowest *r* value being 0.72 between WFDEI and ERA‐Interim (first five rows of Table S1). Only four climate models perform below the exclusion criterion of 0.6 (see Figure 2 left panel and Table 2).

**Figure 3:**
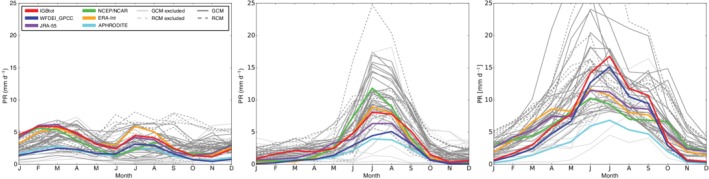
Mean PR yearly cycles of three distinctive regions in the Himalayas between 1981 and 2010 (from east to west, indicated by the green squares in Figure 1). The lines indicate the different observational data sets (colour) and models (grey). Observational data sets are indicated by solid coloured lines, CMIP5 GCMs by solid grey lines and RCMs by dashed grey lines. Models that have been excluded during the PR evaluation are indicated by light grey lines. APHRODITE is showing the lowest PRs of all observational data sets

For precipitation, some models have difficulties simulating the annual cycle (c.f. Figure 3) and obtained correlation coefficients are generally lower than in the case of temperature (see Figure 2 and Table S2). Also the observational uncertainty is higher than for temperature with *r* values ranging between 0.68 and 0.99 (first five rows of Table S2) and again observational disagreements are highest for spatial correlations. While also for PR, climate models show the lowest *r* values for spatial correlations, some models have also deficits in reproducing temporal patterns. Here, our rejection criterion of 0.5 leads to an exclusion of 14 models (2 RCMs) from the subsequent analysis (see right panel in Figure 2 and Table 2). Although we have been focusing on land grid points over the Himalayas only, some of the models removed from our analysis have been identified to perform weak in terms of the Asian and Indian summer monsoon also in other studies (Sperber *et al*., 2013; Sharmila *et al*., 2015, respectively). Precipitation intake from the Indian summer monsoon is most prominent in the eastern Himalayas while WDs are responsible for the bigger part of precipitation in the Hindu Kush and Karakoram (Figure 3).

### 3.2 Elevation‐dependent warming

**Figure 4:**
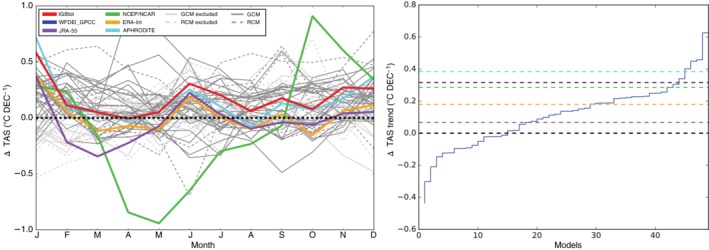
TAS yearly cycle of trend differences between grid points above 3,000 m a.s.l. and lower grid points (<2000 m a.s.l.) (left) between 1981 and 2010. Observational data sets are indicated by solid coloured lines, CMIP5 GCMs by solid grey lines and RCMs by dashed grey lines. Models that did not show positive TAS‐trend differences for DJF by light grey lines. Climate model trend differences between high and low lying grid points of TAS for DJF in °C per decade (right). The culling criterion is marked by the dashed black line, TAS‐trend differences of different observational datasets are marked with dashed coloured lines

On the mentioned 2.5° × 2.5° evaluation grid we calculated differences in TAS linear trends (estimated by ordinary least squares) for the historical period of grid boxes above 3000 m a.s.l. and grid boxes below 2000 m a.s.l.^2^ (c.f. white and red squares in Figure 1, respectively). Trend calculations of reanalysis data can be problematic (e.g., Bengtsson *et al*., 2004) and indeed, throughout the year trend differences between higher and lower altitudes (>3,000 m a.s.l. and <2000 m a.s.l., respectively) varied among the observational data sets (see Figure 4 left panel). Nevertheless, and in agreement with Yan and Liu (2014), all observational data sets showed a higher warming trend at high altitudes for DJF, also for every single month in that season with the exception of JRA55. Throughout the year NCEP/NCAR showed exceptionally high variations of trend differences, while the remaining observational data sets showed a higher level of agreement. Because of the high agreement among all observational data sets for DJF and because of the strong impact of EDW on glaciers, we did not want to abstain from this important aspect of mountain climatology in our evaluation. We therefore excluded models that did not show a higher TAS trend for higher elevations for DJF from the ensemble.

#### 3.2.1 Selection of climate models based on the representation of elevation‐dependent warming during winter

For DJF all observational data sets indicate a higher trend in higher altitudes (cf. Figure 4). More than two thirds of the evaluated climate models were able to reproduce this feature. However, 16 models, out of which 7 are RCMs,^3^ did not reproduce this trend differences and have been removed form the subsequent analysis (see Figure 4 and Table 2). Interestingly, four of five realizations of CCAM‐1391M (see Section 2.3) were not able to reproduce this simplified form of EDW, although EDW signals were present in their driving GCM. In two cases a miss‐representation in the driving GCM lead to a miss‐representation in the RCM as well, while in four cases dynamical downscaling led to an improved representation of EDW. In one case on the other hand, one of three RCMs being driven by the same GCM data has been excluded, while the GCM fulfilled our EDW criterion.

In total we excluded 25 models (8 RCMs^4^) from the ensemble, while 24 models (5 RCMs) remained for the following analysis of climate change over the Indus, Ganga and Brahmaputra river basins (see Table 2 for an overview).

## 4 RESULTS AND DISCUSSION

### 4.1 Projected climate change

**Figure 5:**
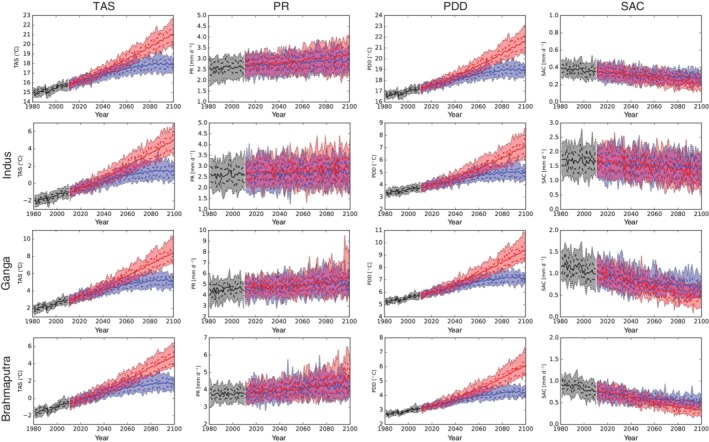
Projected timeseries of mean TAS, PR, PDD and SAC (from left to right respectively) for RCP4.5 (blue) and RCP8.5 (red) for the whole domain and the upper Indus, upper Ganga and upper Brahmaputra catchments (from top to bottom, respectively) of the downsized and bias adjusted ensemble (solid lines). Dashed lines indicate the 25th and 75th percentile, dotted lines the 5th and 95th percentile (shaded area)

**Figure 6:**
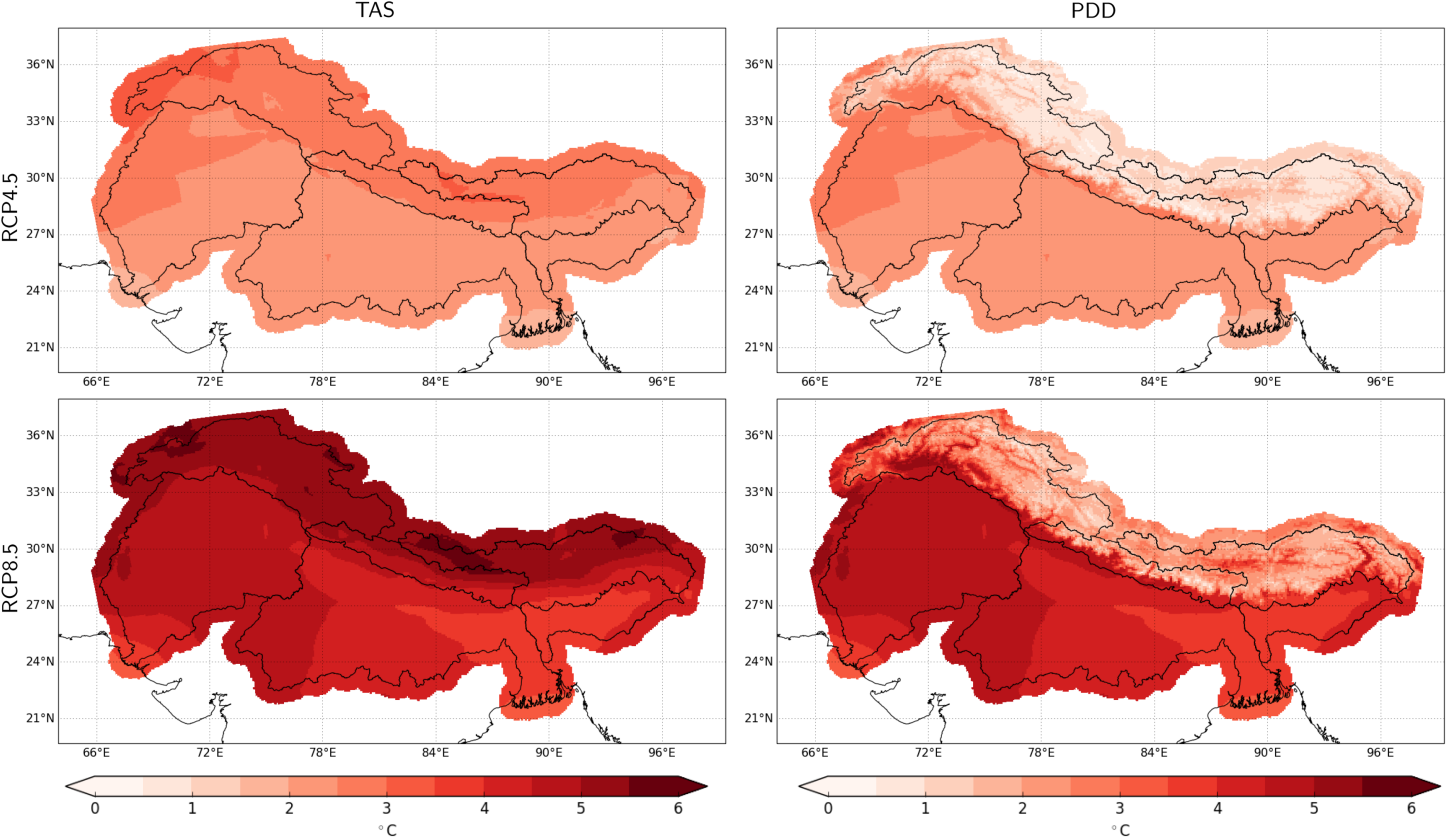
Median projected climate change of annual TAS (left column) and PDD (right column) under the RCP4.5 (top row) and the RCP8.5 (bottom row) scenario until the end of the century (*X*_2071‐2100_ − *X*_1981‐2010_)

**Figure 7:**
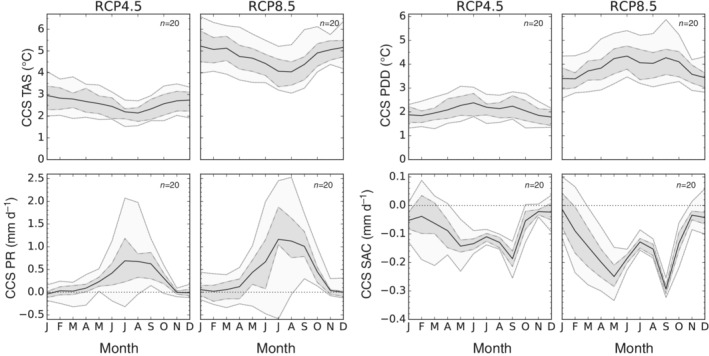
Changes in the annual cycle of TAS (two panels to the top left), PR (two panels to the bottom left), PDD (two panels to the top right) and SAC (two panels to the bottom right) for RCP4.5 (first and third column) and RCP8.5 (second and fourth column) until the end of the century (*X*_2071‐2100_ − *X*_1981‐2010_). The black line indicates the ensemble mean. Dashed lines show the 25th and 75th percentile (grey shaded area), dotted lines the 5th and 95th percentile (light‐grey shaded area). The number in the top right corner of each plot indicates the number of included climate models

Until the end of the century TAS is projected to rise robustly over the entire domain by about ~2.5°C under RCP4.5 and ~5°C under RCP8.5 (Figure 5 top left). The strongest increase in TAS is projected for higher elevations especially under the high emission scenario (Figure 6 left). While there are only small differences in projected temperature changes between the three respective mountainous catchments, there is a tendency of a stronger temperature rise in the West for the three lowland river catchments. In addition, TAS is projected to increase more during winter, though this seasonality is weakly defined (see two panels to the top left in Figure 7).

**Figure 8:**
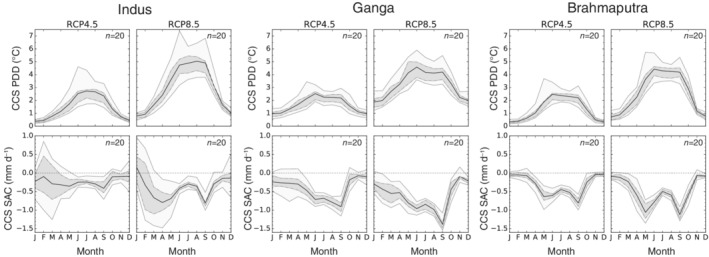
Changes in the annual cycle of PDD (top) and SAC (bottom) for the Indus (left), Ganga (middle) and Brahmaputra (right) upper river basins and the different scenarios (RCP4.5 and RCP8.5) until the end of the century (*X*_2071‐2100_ − *X*_1981‐2010_). The black line indicates the ensemble mean. Dashed lines show the 25th and 75th percentile (grey shaded area), dotted lines the 5th and 95th percentile (light‐grey shaded area). The number in the top right corner of each plot indicates the number of included climate models

For the entire domain and until the end of the century PDD is projected to increase by ~2 and ~4°C under RCP4.5 and RCP8.5, respectively (Figure 7). For the three upstream river catchments projected PDD increases are a little smaller, amounting to ~1.5°C under RCP4.5 and ~3°C under RCP8.5 (Figure 8). However, PDD, the temperature‐based index for glacier melt, behaves opposite to the EDW pattern seen for TAS: the higher the elevation, the lower the projected increase in PDD (c.f. Figure 6). Temperatures, though rising, often stay below the freezing point in high elevations. Likewise, PDD are projected to increase more during summer (see two panels to the top right in Figure 7), especially in the three upstream river catchments (see first row in Figure 8) underpinning the statement that glacier melt will accelerate mostly during summer periods. The projected increase in PDD as well as the increase in TAS are highly significant, all of our included models agree in the sign of projected changes (see also Figures 7 and 8).

**Figure 9:**
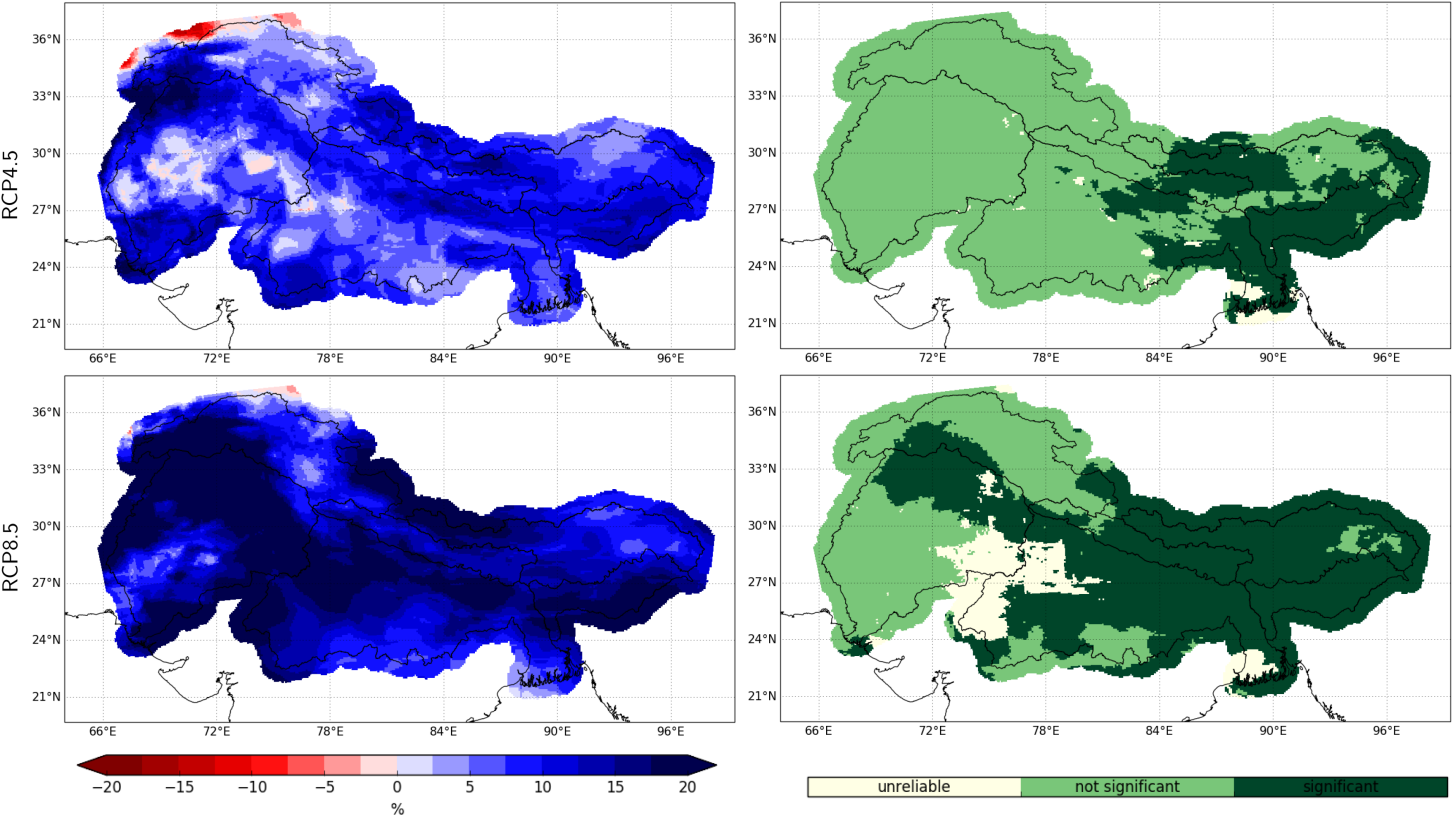
Median climate change of PR for JJAS (left column) under the RCP4.5 (top) and the RCP8.5 (bottom) scenario until the end of the century ((*X*_2071 − 2100_/*X*_1981 − 2010_ − 1) * 100) and the respective levels of agreement between the models (right column; “unreliable”: ≥50% of models show significant changes but agree with <80% on the sign of change; “not significant”: <50% of models show significant changes; “significant”: ≥50% of models show significant changes and agree with ≥80% on the sign of change; significance has been derived using the Wilcoxon‐Mann–Whitney test)

**Figure 10:**
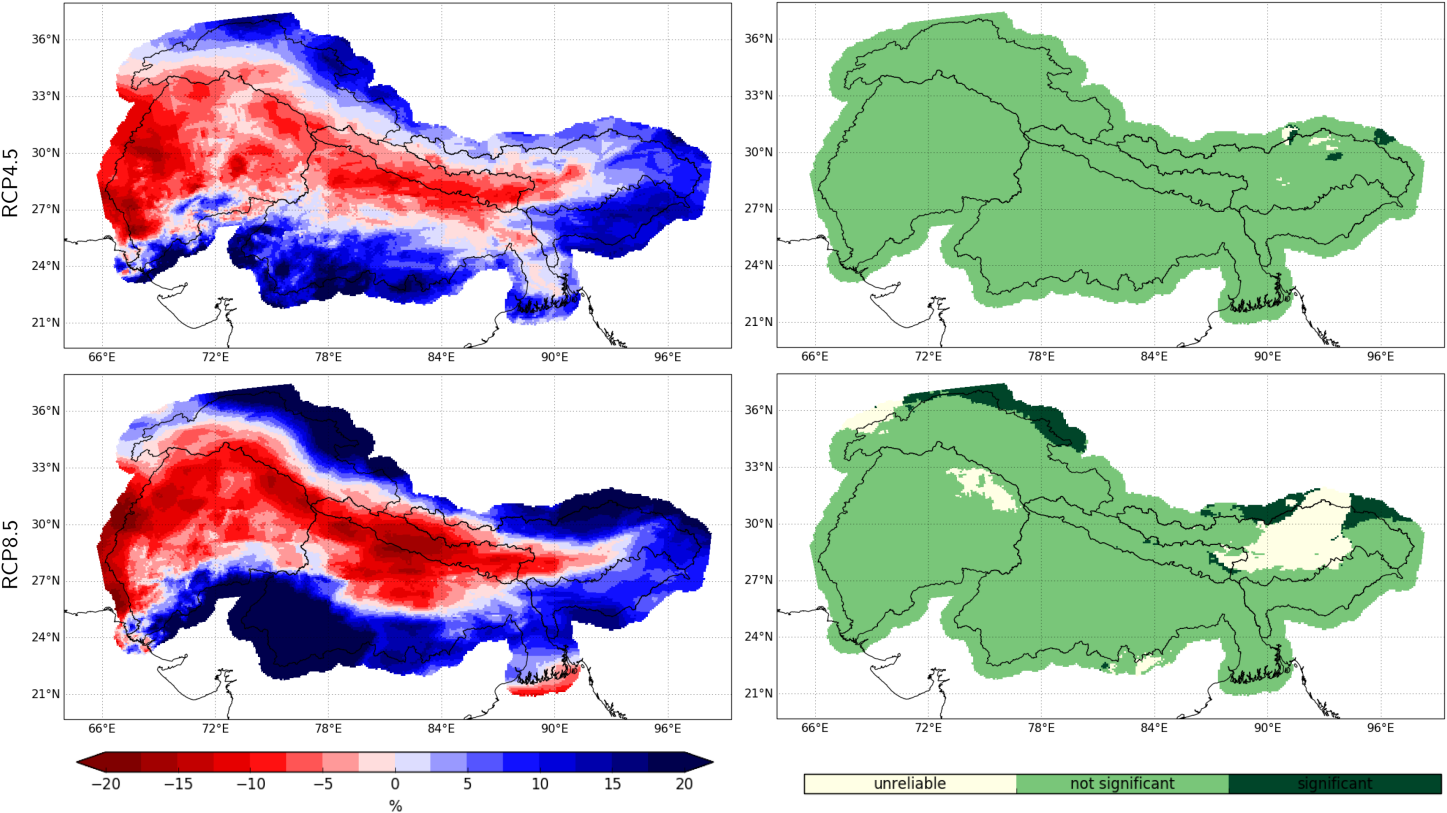
Same as Figure 9 but for projected PR changes for DJFMA

Our downsized bias‐corrected ensemble indicates small increases in mean precipitation for the entire domain and the three upstream river catchments which become larger with progressing time and higher emissions (see Figure 5). However, projected changes in PR show a heterogeneous spatio‐temporal behaviour. Until the end of the century increases are expected in particular during the ISM period (two panels to the bottom left in Figure 7). This increases during JJAS are especially pronounced over the eastern and central Himalayas (Brahmaputra and Ganga catchments, Figures 9 and S3), where the models agree also on the sign of the projected JJAS changes under RCP4.5. For the higher emission scenario RCP8.5 the models additionally project significant precipitation increases over the Indus catchment during the ISM (c.f. Figure 9). Contrary to the projected increase in monsoon precipitation, there is a tendency of decreasing WDs dominated DJFMA precipitation for the Ganga high elevation catchment as well as large parts of the Indus and Ganga lowlands (c.f. Figure 10). For the higher emission scenario this signal enhances further. Significant changes during DJFMA are projected only for the northern boundaries of the research domain, being the northern areas of the Indus and Brahmaputra high elevation catchments. The contrasting behaviour of robustly increasing precipitation rates during summer and uncertain projections during winter is also visible in the seasonal cycle over the entire domain (Figure 7 two panels to the bottom left). However, while the increase in JJAS PR in relative terms is clearly indicated in the map plots (Figure 9), increasing absolute PR values are hardly reflected in the seasonal cycle over the arid upper Indus catchment (Figure S3).

**Figure 11:**
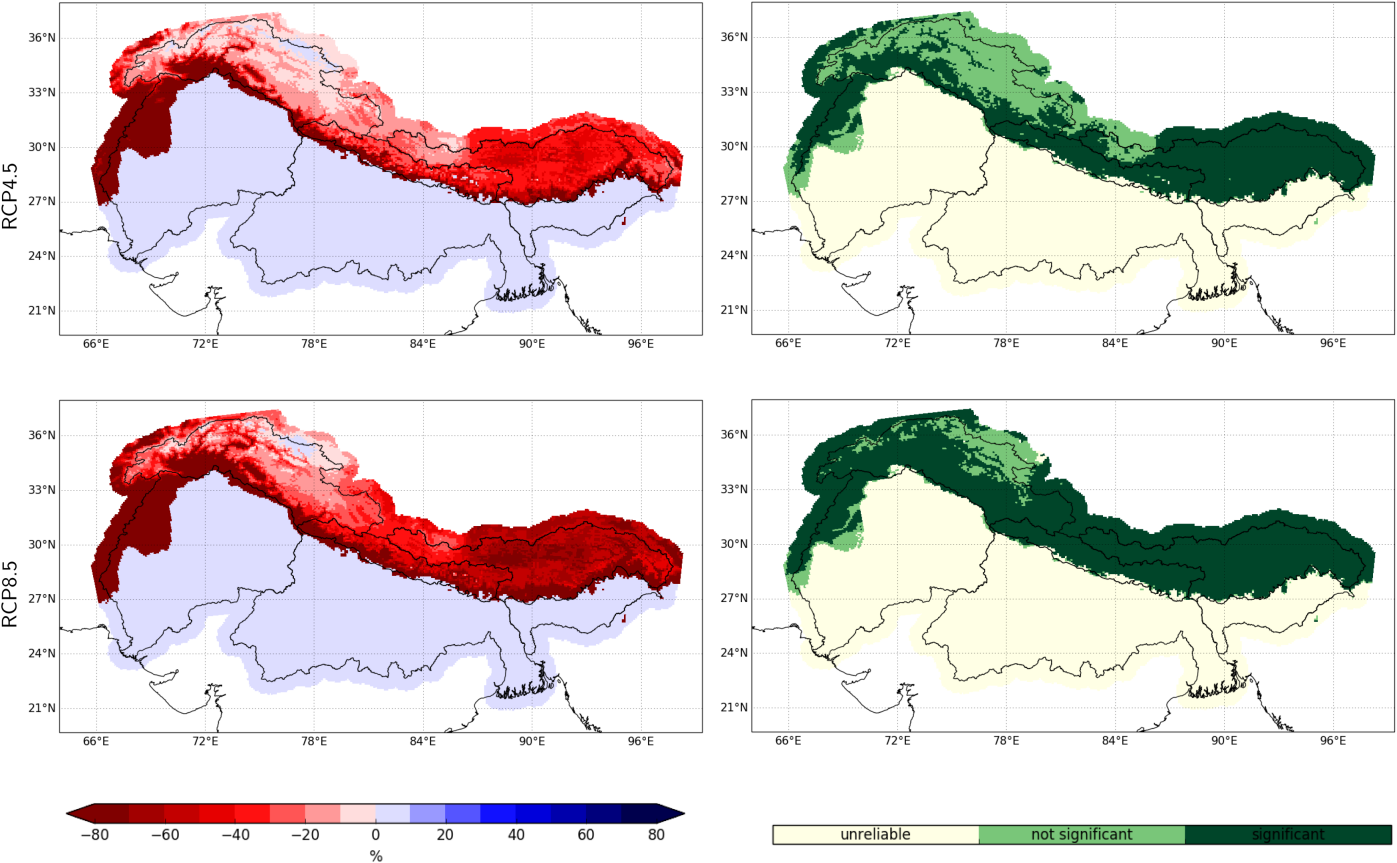
Median climate change of SAC (left column) under the RCP4.5 (top) and the RCP8.5 (bottom) scenario until the end of the century ((*X*_2071 − 2100_/*X*_1981 − 2010_ − 1) * 100) and the respective levels of agreement between the models (right column; “unreliable”: ≥50% of models show significant changes but agree with <80% on the sign of change; “not significant”: <50% of models show significant changes; “significant”: ≥50% of models show significant changes and agree with ≥80% on the sign of change; significance has been derived using the Wilcoxon‐Mann–Whitney test)

In contrast to PR, the derived SAC index is projected to decrease significantly, especially for longer timescales and the higher emission scenario (see Figures 5 and 11). The largest decreases are displayed for the transitional months May and September (c.f. Figure 7), where large parts of former solid precipitation are projected to precipitate in liquid form in the future. Especially over the eastern catchments a larger decline in SAC is projected (see bottom row in Figure 8). Over the upper Indus catchment however model disagreement in projected SAC changes is high (c.f. Figure 11 right), and in the winter months the sign of the change is not clear at all (see Figure 8 bottom left). The contrasting behaviour of PR and SAC during the seasons when WDs and the ISM predominate is evident in their temporal evolution (see Figure S2). PR and SAC during DJFMA are projected to change little with progressing time. On the other hand, increases in JJAS PR are accompanied by higher temperatures and go along with stronger decreases in SAC with increasing time and higher emissions.

The projected changes will likely have strong impacts for the regional cryosphere and hydrology throughout the 21st century. The projected increase in PDD and decrease in SAC imply accelerated melt and eventual reduction of ice volumes. The large range in the projections also implies a large uncertainty in the timing and magnitude of the impacts for cryosphere and hydrology. For example, Kraaijenbrink *et al*. (2017) projected end of century glacier volume reductions in the HMA region varying from 15 to 75% by forcing a glacier model with all climate models in CMIP5 for all four RCPs. Several regional or global scale studies (Lutz *et al*., 2016; Huss and Hock, 2018; Zheng *et al*., 2018) showed strong variability in the timing of hydrological response to future glacier changes. The use of our data set to force these types of models would most likely result in similar trends, however, it could narrow down the uncertainty resulting from the climate model ensemble range and lead to more robust projections.

### 4.2 Projected changes in EDW signals

**Figure 12:**
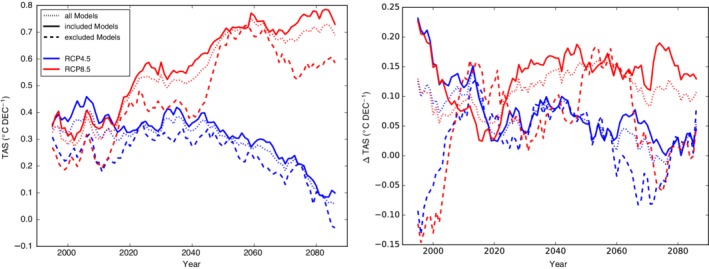
Evolution of DJF TAS trends over all evaluation grid‐points (left) and of TAS trend differences (Δ*TAS*) of grid‐points above 3,000 m a.s.l. and below 2000 m a.s.l. (right) for all models (dotted lines), models that have not been (solid lines) and models that have been excluded (dashed lines) during the EDW evaluation. Trends have been calculated for a period of 29 years centered around the indicated year

Figure 12 shows the evolution of mean DJF TAS trends for all evaluation grid‐points (white and red boxes in Figure 1) and differences in trends between grid‐points above 3,000 m a.s.l. and below 2000 m a.s.l. of all models, models that have and models that have not been excluded during the EDW evaluation. RCP4.5 TAS trends remain virtually constant over the first half of the 21st century and with stabilizing CO_2_ concentrations converge towards zero towards the end of the century. Conversely, RCP8.5 TAS trends show a steady increase until 2050 and remain virtually constant afterwards (Figure 12 left). At the same time trend differences between low lying areas and the high mountain environment (i.e., our evaluation criterion of EDW) reflect the additional warming under RCP8.5 and hence higher EDW by higher trend differences in comparison to RCP4.5 in particular from 2020 onward until the end of the century (Figure 12 right).

Excluded models in Figure 12 are models that did not show positive trend differences during DJF over the evaluation period (*∅* 1981–2010), where our observational data sets indicated trend differences between 0.07 and 0.32°C per decade (see Subsection 3.2). Notably, the mean EDW signal of not excluded (i.e., included in Figure 12) models stays well above the mean EDW signal of excluded models until around 2010 (*∅* 1996–2024). Contrastingly, thereafter and until around 2020 excluded models indicate slightly higher EDW than included models. Even though excluded models show EDW, this later EDW onset suggests a mistiming of EDW relevant processes in the excluded models. From 2020 onwards the two ensembles (included and excluded) show similar EDW signals for RCP4.5 with a tendency of higher EDW reported by the included models, EDW signals of the two ensembles differ stronger for RCP8.5 also with higher EDW shown by the included models. In addition to the mean negative DJF EDW signal of excluded models until the beginning of the 21st century, there are episodes of negative EDW signals under the forcing of both emission scenarios around 2070.

Also, the two ensembles show differences in absolute TAS trends. Models that did show higher rates of EDW also report higher rates of warming throughout the 21st century. Differences are larger for the high emission scenario RCP8.5. While there is some similarity between the curves of TAS trends and TAS trend differences, the very large differences in the EDW signal until 2010 between the two ensembles is not strongly reflected in the TAS trends.

## 5 SUMMARY AND CONCLUSIONS

In this study, we have combined GCMs and RCMs to one consistent climate model ensemble, and downsized this ensemble by removing models that were not able to reproduce important weather features over the HKKH region and a simplified form of EDW. We subsequently bias corrected the remaining models with a trend preserving error correction method to provide climate scenarios over the Indus, Ganga and Bramaputhra river catchments. As glaciers play a key role in hydrological modelling efforts over high mountain environments, presented results have been focusing on changes in PDD and SAC in addition to TAS and PR. The resulting data set can be used as forcing for glacio‐hydrological modelling to generate region‐specific robust projections of glacier change and hydrological changes which can inform on climate change impacts in a wide range of sectors, and is available upon request.

TAS is projected to rise robustly by about ~2.5°C under RCP4.5 and ~5°C under RCP8.5 until the end of the century, while we found an intensified warming for higher altitudes. At the same time also PDD is robustly projected to increase, with opposite effects for higher altitudes. Chaturvedi *et al*. (2012) reported similar findings over India. In addition, our model ensemble indicates a robust rise of PR connected to the ISM over the entire research domain, while PR connected to WDs is projected to decrease over the southern Himalayan foreland and the Ganga catchment for high elevations. Projections for PR are in general found to be both, more robust and stronger for the higher emission scenario. The intensifications shown by the ensemble mean in terms of ISM PR are in agreement with remarks in literature (Chaturvedi *et al*., 2012; Sharmila *et al*., 2015). Regardless of the overall increase in PR, SAC is projected to decrease robustly.

Since we evaluated models in terms of their representation of EDW, and included models are found to exhibit a stronger climate sensitivity, our downsized ensemble shows higher warming rates, than the mean over all models (included and excluded). While it certainly is possible to argue that this approach disagrees with the often applied one‐model‐one‐vote practice, we find it fitting to exclude models for the generation of user‐focused impact scenarios that do not feature observable phenomena correctly. In addition, the onset, timing and perpetuation of EDW can be seen in agreement with the concept of emergent constraints (Collins *et al*., 2012).

## Supporting information




**Figure S1** Same as Figure 3 but for TAS

**Figure S2** Same as Figure 5 but for DJFMA PR and SAC (two columns to the left) and JJAS PR and SAC (two columns to the right)

**Figure S3** Same as Figure 8 but for TAS (top) and PR (bottom)

**Table S1** Pearson correlations of TAS on the Himalayan southern ridge. Columns denote temporal (time) spatial (gp) and combined correlations to TAS in the observational datasets (IGB: I, WFDEI GPCC: W, JRA55: J, NCEP/NCAR: N, ERA‐Interim: E). Rows denote the ensemble (Hybrid: Hy; Reanalysis: Re; CMIP5: CM; SA‐CORDEX: SA, EA‐CORDEX: EA), the GCM and the model run, as well as possibly the RCM. Excluded models are marked with an “X” at the beginning, followed by the crucial culling criterion(s) (tr: TAS correlations; pr: PR correlations; tt: TAS trend differences). The cell colour corresponds to the strength of the relationship (yellowish [strong] to reddish [weak])

**Table S2** Same as Table S1 but for PR

## Endnotes

1 Some of the SA‐CORDEX are not limited area RCMs per definition. The Conformal Cubic Atmospheric Models (CCAMs) were developed by the Commonwealth Scientific and Industrial Research Organization (CSIRO), Australia. The CCAM is a variable–resolution global atmospheric model with enhanced resolution over specific regions to simulate the regional scenarios not using lateral boundary conditions. Bias and variance corrected sea surface temperatures (SSTs) of global climate projections were used to simulate the regional atmosphere by the CCAMs. Five regional simulations of CCAM‐1391 M were used in this study.

2 The elevation for the evaluated grid points was calculated using the DEM of IGB (SRTM DEM, Farr *et al*., 2007), although inter‐model spread of underlying elevation data between models is substantial, sometimes even as high as 500 m (cf. Kotlarski *et al*., 2015; Palazzi *et al*., 2017). We justify this generalization of model height because our compared evaluation grid points for high and low elevations differ by at least 1,000 m.

3 Including 4 realizations of the CCAM‐1391 M model.

4 Including all 5 realizations of the CCAM‐1391 M model.
