# Supplementary figures and images for: Climate projections for glacier change modelling over the Himalayas
Source: Int J Climatol. 2019 Dec 25;40(3):1738–54. doi: 10.1002/joc.6298 (PMC7078814; doi:10.1002/joc.6298)

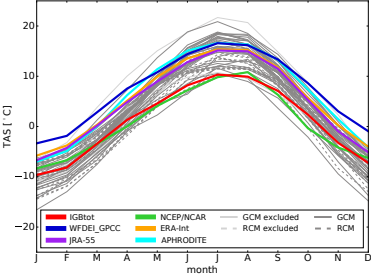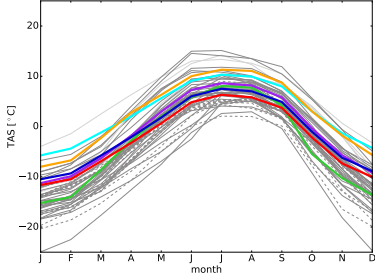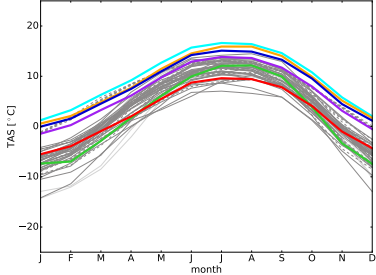

Supplement: Supplementary file 1 — Figure S1 Same as Figure 3 but for TAS [file JOC-40-1738-s001.pdf]

DJFMA

JJAS

PR

SAC

PR

SAC

Indus

# Ganga

# Brahmaputra

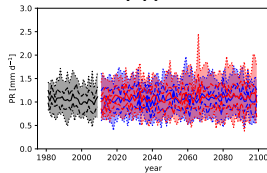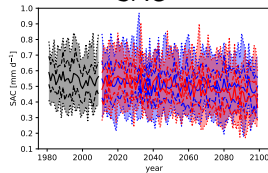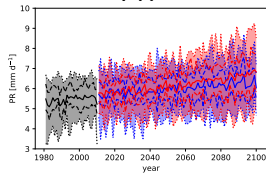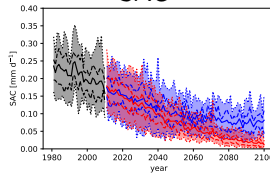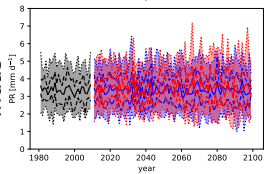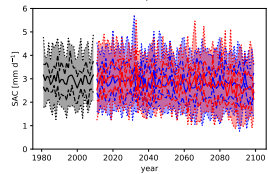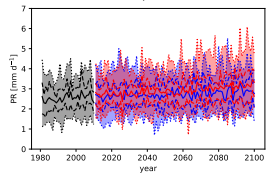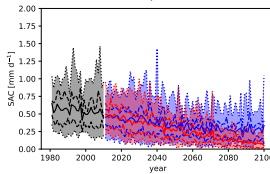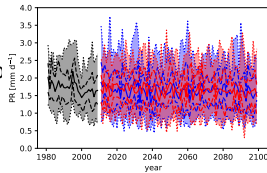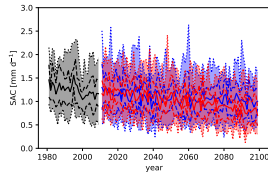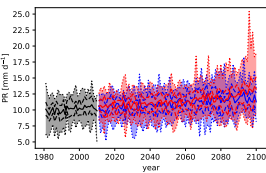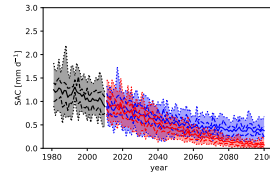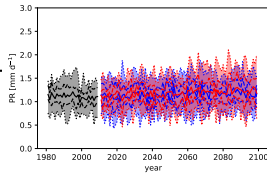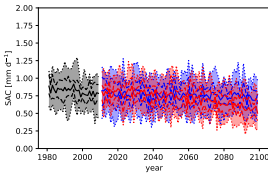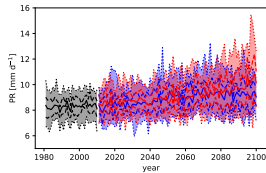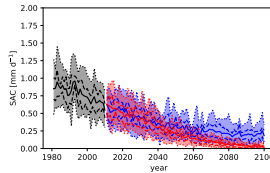

Supplement: Supplementary file 2 — Figure S2 Same as Figure 5 but for DJFMA PR and SAC (two columns to the left) and JJAS PR and SAC (two columns to the right) [file JOC-40-1738-s002.pdf]

## Indus

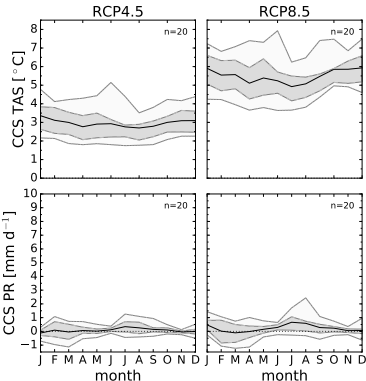

## Ganga

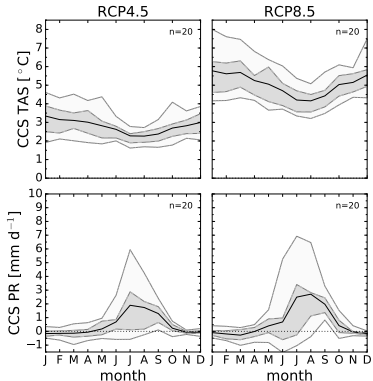

## Brahmaputra

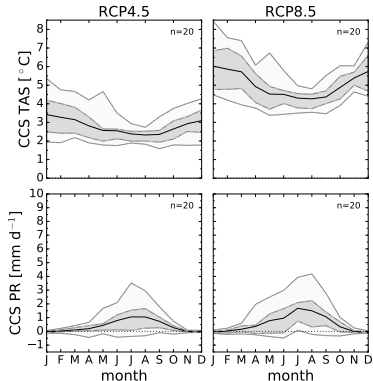

Supplement: Supplementary file 3 — Figure S3 Same as Figure 8 but for TAS (top) and PR (bottom) [file JOC-40-1738-s003.pdf]
