# Supplementary material for: Climate projections for glacier change modelling over the Himalayas
Source: Int J Climatol. 2019 Dec 25;40(3):1738–54. doi: 10.1002/joc.6298 (PMC7078814; doi:10.1002/joc.6298)
Supplement: Supplementary file 5 — Table S2 Same as Table S1 but for PR [file JOC-40-1738-s005.pdf]

	I pr time r	I pr gp r	I pr r	W pr time r	W pr gp r	W pr r	J pr time r	J pr gp r	J pr r	N pr time r	N pr gp r	N pr r	E pr time r	E pr gp r	E pr r
Hy IGBtot	1.00	1.00	1.00	0.99	0.91	0.95	0.97	0.86	0.92	0.93	0.80	0.87	0.94	0.68	0.84
Re WFDEI_GPCC	0.99	0.91	0.95	1.00	1.00	1.00	0.95	0.88	0.90	0.87	0.82	0.80	0.94	0.78	0.79
Re JRA-55	0.97	0.86	0.92	0.95	0.88	0.90	1.00	1.00	1.00	0.94	0.88	0.89	0.97	0.90	0.89
Re NCEP/NCAR	0.93	0.80	0.87	0.87	0.82	0.80	0.94	0.88	0.89	1.00	1.00	1.00	0.93	0.79	0.86
Re ERA-Int	0.94	0.68	0.84	0.94	0.78	0.79	0.97	0.90	0.89	0.93	0.79	0.86	1.00	1.00	1.00
CM ACCESS1-0 r1i1p1	0.84	0.72	0.82	0.83	0.83	0.77	0.89	0.87	0.88	0.83	0.82	0.85	0.93	0.87	0.91
X tt SA ACCESS1-0 r1i1p1 CSIRO CCAM-1391M	0.65	0.77	0.71	0.71	0.81	0.78	0.70	0.76	0.74	0.69	0.69	0.67	0.73	0.51	0.53
CM ACCESS1-3 r1i1p1	0.87	0.61	0.82	0.88	0.74	0.80	0.91	0.83	0.88	0.82	0.73	0.80	0.94	0.88	0.93
X pr tt CM bcc-csm1-1 r1i1p1	0.49	0.22	0.54	0.33	0.00	0.51	0.43	0.11	0.49	0.45	0.20	0.52	0.29	0.30	0.59
CM bcc-csm1-1-m r1i1p1	0.90	0.72	0.82	0.91	0.60	0.78	0.85	0.72	0.81	0.82	0.80	0.87	0.84	0.67	0.84
X pr CM BNU-ESM r1i1p1	0.85	0.34	0.70	0.81	0.13	0.63	0.80	0.38	0.67	0.77	0.57	0.76	0.72	0.54	0.72
CM CanESM2 r1i1p1	0.79	0.56	0.73	0.78	0.58	0.72	0.73	0.70	0.72	0.72	0.77	0.73	0.66	0.78	0.81
CM CCSM4 r1i1p1	0.89	0.84	0.86	0.93	0.87	0.88	0.87	0.89	0.88	0.91	0.88	0.87	0.90	0.71	0.78
CM CESM1-BGC r1i1p1	0.89	0.83	0.85	0.92	0.87	0.88	0.89	0.90	0.88	0.89	0.88	0.86	0.90	0.75	0.79
CM CESM1-CAM5 r1i1p1	0.92	0.80	0.83	0.95	0.85	0.87	0.93	0.91	0.90	0.93	0.86	0.86	0.95	0.76	0.82
X tr pr CM CMCC-CESM r1i1p1	0.83	0.37	0.44	0.76	0.02	0.31	0.84	0.33	0.47	0.88	0.37	0.60	0.84	0.52	0.69
CM CMCC-CM r1i1p1	0.90	0.87	0.83	0.87	0.91	0.85	0.85	0.87	0.85	0.84	0.81	0.78	0.79	0.80	0.78
CM CMCC-CMS r1i1p1	0.91	0.78	0.80	0.86	0.70	0.74	0.86	0.82	0.81	0.87	0.87	0.85	0.82	0.82	0.84
CM CNRM-CM5 r1i1p1	0.93	0.86	0.88	0.88	0.81	0.85	0.92	0.90	0.91	0.89	0.91	0.88	0.89	0.79	0.84
X tt SA CNRM-CM5 r1i1p1 CSIRO CCAM-1391M	0.64	0.73	0.69	0.73	0.80	0.77	0.71	0.75	0.73	0.66	0.68	0.67	0.74	0.53	0.54
X pr CM CSIRO-Mk3-6-0 r1i1p1	0.79	0.54	0.68	0.72	0.42	0.64	0.73	0.58	0.67	0.73	0.67	0.72	0.68	0.68	0.76
X tr pr CM CSIRO-Mk3L-1-2 r1i2p1	0.67	0.00	0.54	0.61	0.00	0.47	0.62	0.00	0.49	0.55	0.00	0.51	0.51	0.02	0.57
EA EC-EARTH r3i1p1 DMI HIRHAM5	0.93	0.86	0.86	0.94	0.95	0.89	0.91	0.91	0.88	0.93	0.85	0.84	0.94	0.77	0.73
X tt CM EC-EARTH r12i1p1	0.91	0.81	0.90	0.93	0.90	0.91	0.92	0.96	0.92	0.91	0.89	0.86	0.95	0.84	0.85
X tt SA EC-EARTH r12i1p1 SMHI RCA4	0.93	0.86	0.88	0.93	0.91	0.89	0.92	0.90	0.90	0.90	0.88	0.90	0.92	0.79	0.82
CM GFDL-CM3 r1i1p1	0.80	0.65	0.68	0.78	0.62	0.64	0.75	0.78	0.67	0.71	0.81	0.69	0.66	0.85	0.77
X tt SA GFDL-CM3 r1i1p1 CSIRO CCAM-1391M	0.65	0.76	0.69	0.72	0.81	0.77	0.72	0.75	0.73	0.65	0.69	0.66	0.72	0.51	0.52
X tt CM GFDL-ESM2G r1i1p1	0.89	0.68	0.79	0.88	0.53	0.72	0.82	0.68	0.75	0.83	0.85	0.84	0.80	0.70	0.80
X tt CM GFDL-ESM2M r1i1p1	0.89	0.62	0.79	0.92	0.58	0.74	0.80	0.71	0.76	0.79	0.85	0.82	0.80	0.75	0.82
X pr CM GISS-E2-H r6i1p3	0.55	0.79	0.54	0.41	0.64	0.51	0.51	0.76	0.57	0.46	0.70	0.44	0.39	0.70	0.55
X pr tt CM GISS-E2-R r6i1p1	0.42	0.78	0.37	0.21	0.61	0.32	0.45	0.73	0.42	0.37	0.59	0.27	0.32	0.63	0.32
X tt CM HadGEM2-AO r1i1p1	0.89	0.71	0.85	0.89	0.82	0.79	0.92	0.86	0.88	0.86	0.84	0.86	0.95	0.86	0.91
X tt EA HadGEM2-AO r1i1p1 NIMR HadGEM3-RA	0.92	0.85	0.81	0.89	0.95	0.84	0.94	0.92	0.91	0.90	0.81	0.79	0.95	0.82	0.81
EA HadGEM2-AO r1i1p1 SNU MM5	0.83	0.92	0.84	0.76	0.81	0.81	0.80	0.85	0.83	0.75	0.82	0.79	0.72	0.76	0.78
EA HadGEM2-AO r1i1p1 SNU WRF	0.94	0.81	0.85	0.91	0.73	0.78	0.93	0.80	0.83	0.86	0.80	0.85	0.90	0.90	0.90
EA HadGEM2-AO r1i1p1 KNU RegCM4	0.89	0.82	0.82	0.85	0.86	0.82	0.87	0.88	0.85	0.83	0.83	0.81	0.83	0.83	0.85
CM HadGEM2-CC r1i1p1	0.86	0.69	0.82	0.86	0.81	0.76	0.91	0.87	0.86	0.85	0.83	0.85	0.94	0.87	0.90
CM HadGEM2-ES r1i1p1	0.87	0.72	0.83	0.87	0.80	0.76	0.92	0.88	0.86	0.85	0.83	0.85	0.94	0.85	0.88
X tt CM inmcm4 r1i1p1	0.89	0.78	0.80	0.86	0.60	0.72	0.85	0.71	0.77	0.81	0.79	0.82	0.79	0.72	0.80
X pr tt CM IPSL-CM5A-LR r1i1p1	0.23	0.44	0.23	0.03	0.39	0.16	0.21	0.45	0.28	0.22	0.49	0.27	0.03	0.38	0.17
X pr CM IPSL-CM5A-MR r1i1p1	0.66	0.73	0.69	0.57	0.80	0.72	0.63	0.85	0.74	0.61	0.83	0.70	0.50	0.83	0.70
X pr tt CM IPSL-CM5B-LR r1i1p1	0.00	0.31	0.00	0.00	0.29	0.00	0.00	0.32	0.00	0.00	0.27	0.00	0.00	0.35	0.00
X tr pr CM MIROC-ESM r1i1p1	0.77	0.43	0.72	0.84	0.19	0.67	0.75	0.27	0.63	0.68	0.38	0.65	0.79	0.22	0.69
X tr pr CM MIROC-ESM-CHEM r1i1p1	0.81	0.39	0.75	0.87	0.19	0.71	0.79	0.27	0.67	0.73	0.37	0.68	0.83	0.22	0.72
CM MIROC5 r1i1p1	0.96	0.82	0.87	0.91	0.77	0.82	0.96	0.85	0.89	0.95	0.90	0.92	0.93	0.80	0.85
CM MPI-ESM-LR r1i1p1	0.95	0.74	0.85	0.91	0.67	0.79	0.93	0.84	0.85	0.95	0.90	0.91	0.93	0.82	0.88
SA MPI-ESM-LR r1i1p1 MPI-CSC REMO2009	0.93	0.84	0.81	0.91	0.91	0.83	0.91	0.88	0.88	0.90	0.85	0.85	0.91	0.78	0.80
X tt SA MPI-ESM-LR r1i1p1 IAU CCLM	0.88	0.87	0.85	0.84	0.88	0.84	0.90	0.91	0.91	0.89	0.87	0.88	0.91	0.83	0.88
X pr SA MPI-ESM-LR r1i1p1 CSIRO CCAM-1391M	0.63	0.75	0.71	0.70	0.80	0.78	0.69	0.74	0.74	0.69	0.68	0.68	0.72	0.49	0.53
CM MPI-ESM-MR r1i1p1	0.95	0.79	0.86	0.91	0.70	0.80	0.93	0.85	0.86	0.94	0.90	0.91	0.90	0.83	0.88
CM MRI-CGCM3 r1i1p1	0.75	0.80	0.75	0.73	0.77	0.73	0.73	0.83	0.77	0.67	0.83	0.76	0.72	0.80	0.83
CM MRI-ESM1 r1i1p1	0.71	0.80	0.74	0.68	0.76	0.72	0.70	0.81	0.76	0.66	0.82	0.76	0.70	0.79	0.82
CM NorESM1-M r1i1p1	0.89	0.74	0.84	0.89	0.57	0.78	0.84	0.71	0.81	0.84	0.80	0.84	0.81	0.65	0.79
X pr SA NorESM1-M r1i1p1 CSIRO CCAM-1391M	0.67	0.73	0.70	0.73	0.80	0.78	0.73	0.75	0.74	0.67	0.68	0.66	0.74	0.50	0.52
